# Supplementary material for: LTα, TNF, and ILC3 in Peyer’s Patch Organogenesis
Source: Cells. 2022 Jun 19;11(12):1970. doi: 10.3390/cells11121970 (PMC9221848; doi:10.3390/cells11121970)
Supplement: Supplementary file 1 [file cells-11-01970-s001.zip › cells-1739417-supplementary.pdf]

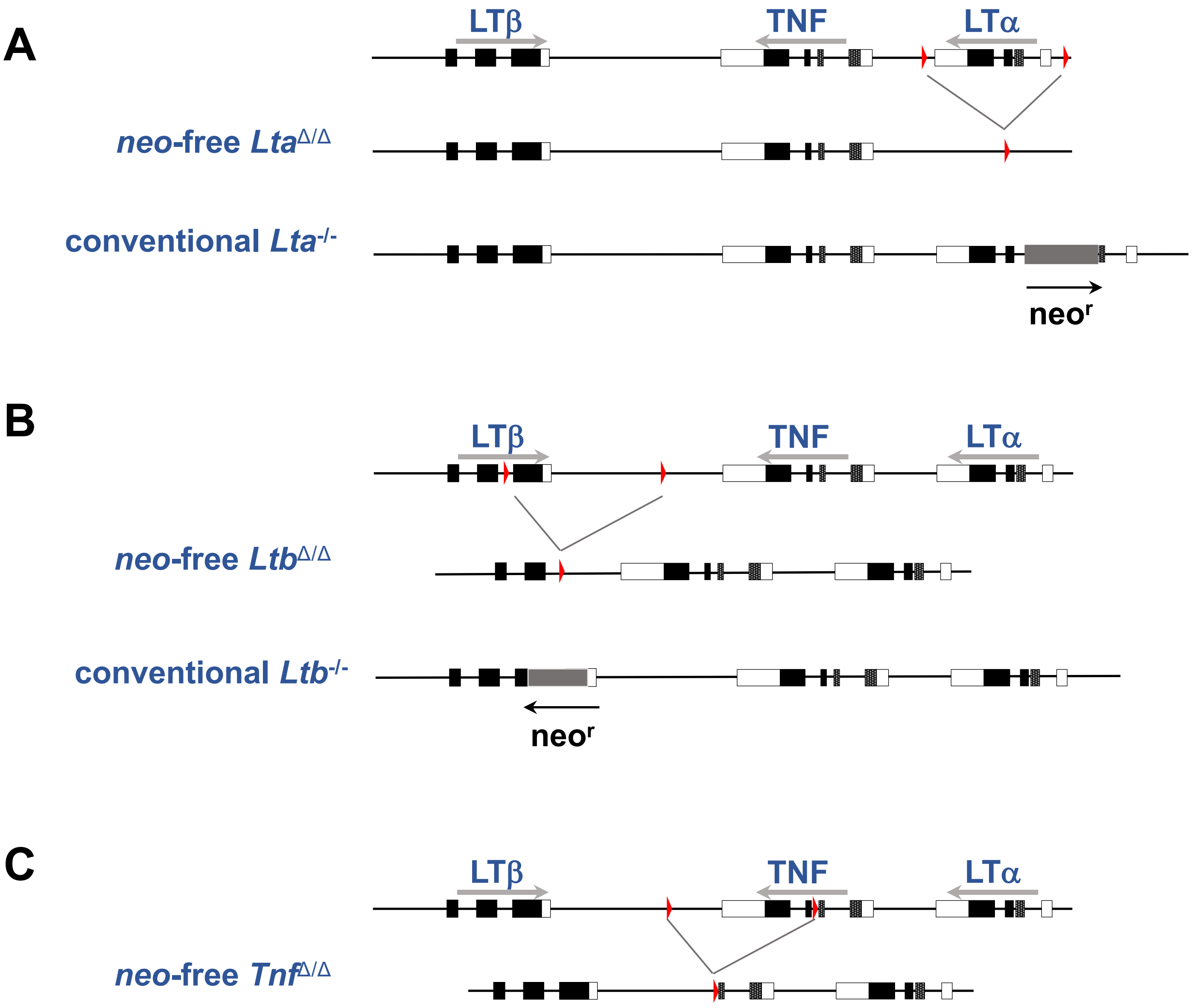

Figure S1: Comparison of different LTα, LTβ and TNF genes targeting strategies. Schematic rep-resentation of wild-type TNF/LT locus and (**A**) *neo*-free and conventional *Lta*-deficient mice, (**B**) *neo*-free and conventional *Ltb*-deficient mice, (**C**) *neo*-free *Tnf*-deficient mice.

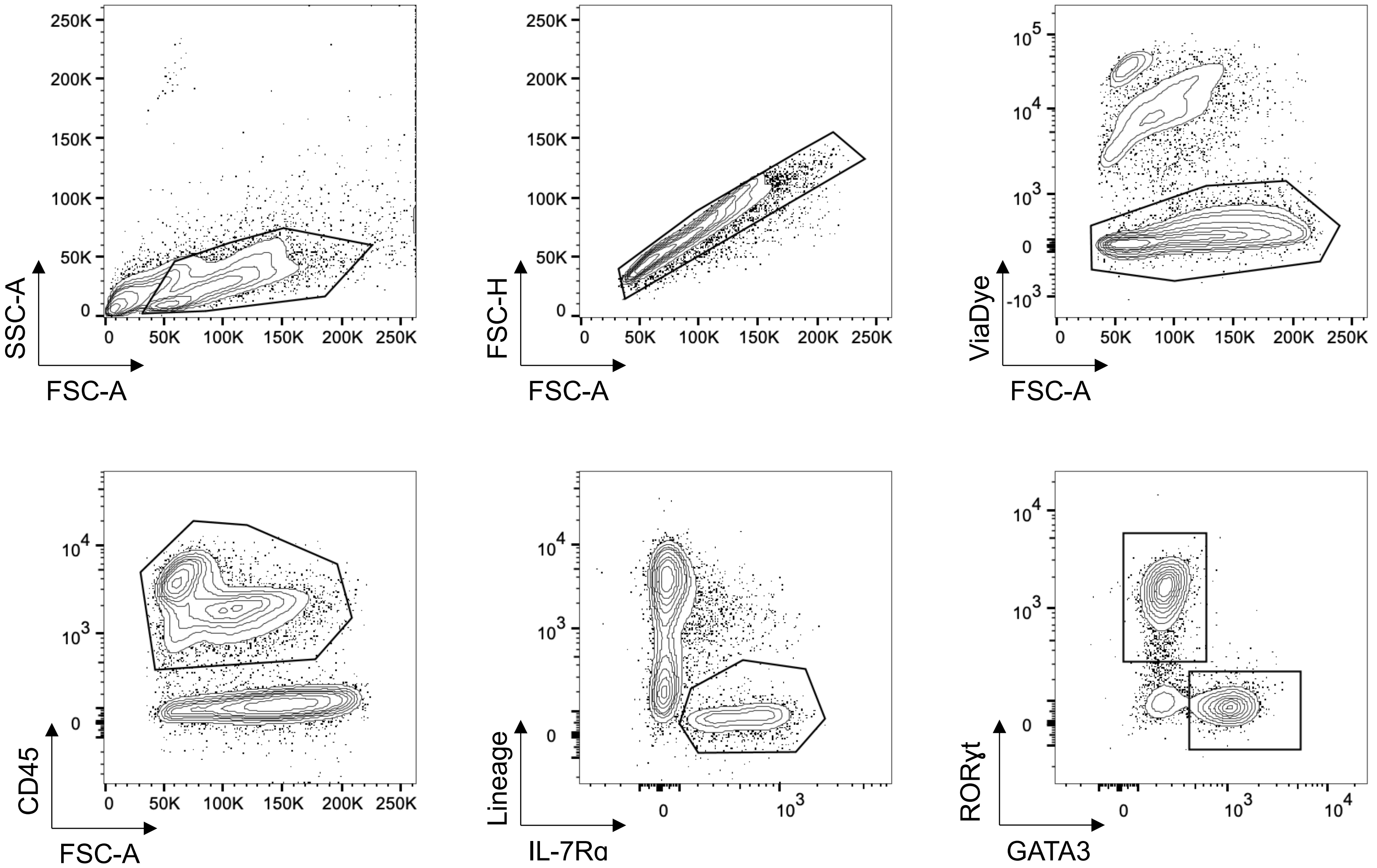

Figure S2: Flow cy-tometry gating strategy for identification of ILC subsets in the lamina propria of the small intestine. ILC3 were identified as ViaDye^{-}CD45+Lin^{-} IL-7Rα^{+}RORγt^{+} cells.
